# Sarcomatoid hepatocellular carcinoma with subdiaphragmatic metastasis misdiagnosed as liver abscess: A case report

**DOI:** 10.1097/MD.0000000000040842

**Published:** 2024-12-06

**Authors:** Xue-Wu Liu, Chun-Yuan Yang, Xue-Ping Liu, Nan Lei

**Affiliations:** a Radiology Department, the People’s Hospital of Lezhi, Ziyang, Lezhi, China; b Department of Hepatobiliary and Pancreatic Surgery, the People’s Hospital of Lezhi, Ziyang, Lezhi, China; c Pathology Department, the People’s Hospital of Lezhi, Ziyang, Lezhi, China.

**Keywords:** case report, liver abscess, mis-diagnose, sarcomatoid hepatocellular carcinoma

## Abstract

**Rationale:**

Sarcomatoid hepatocellular carcinoma (SHC) is a rare subtype of hepatocellular carcinoma. Its imaging findings often resemble those of liver abscess, making preoperative diagnosis particularly challenging. To date, there have been no documented cases of SHC with subdiaphragmatic metastases. In this report, we present a case of SHC with subdiaphragmatic metastasis that was initially misdiagnosed as hepatic abscess. In addition, we conducted a retrospective analysis of the clinical and imaging findings to improve the clinical understanding of this disease.

**Patient concerns:**

A 74-year-old woman was admitted to our hospital with recurrent right upper abdominal pain and discomfort, chills, and fever for >1 month.

**Diagnoses:**

The patient underwent abdominal computed tomography and magnetic resonance imaging, which revealed multiple nodules and masses in the left lobe of the liver. Furthermore, a thick-walled irregular cystic solid mass was identified in the anterior and subdiaphragmatic regions. Based on these findings, the patient was diagnosed with an abscess. The postoperative pathology confirmed SHC in both the left lobe of the liver and subdiaphragmatic mass.

**Interventions:**

The patient underwent a left external liver lobectomy.

**Outcomes:**

The patient’s condition deteriorated after surgery, and hepatic encephalopathy developed 1.5 months postoperation, ultimately leading to death.

**Lessons:**

To the best of our knowledge, cases of SHC with subdiaphragmatic metastases are rare, and the preoperative diagnosis presents a significant challenge in clinical practice. More comprehensive case analyses of SHC are needed to enhance the accuracy of clinical and imaging diagnoses.

## 1. Introduction

Sarcomatoid hepatocellular carcinoma (SHC), also known as spindle cell carcinoma, is a rare malignant tumor with a sarcomatoid component. It is a pathological subtype of primary liver cancer, accounting for 1.8% to 3.9% of hepatocellular carcinoma.^[1]^ Most scholars believe that SHC is usually secondary to repeated invasive treatments of hepatic tumors, such as conventional transcatheter arterial chemoembolization, anhydrous alcohol injection, and microwave ablation, with primary SHC being rare.^[2,3]^

Liver abscess is a suppurative infection of the liver parenchyma caused by pathogenic bacteria through various channels, with bacterial liver abscess being the most common form.^[4]^ The clinical symptoms and imaging findings of SHC are often similar to those of liver abscess, leading to difficulties in preoperative diagnosis and treatment delays.^[5]^

Here, we report a case of SHC with subdiaphragmatic metastasis in a patient who had not previously received treatment for a liver tumor. Preoperative imaging revealed that a part of the lesion was misdiagnosed as a liver abscess.

## 2. Case presentation

In May 2024, a 74-year-old woman was admitted to our hospital with recurrent right upper abdominal pain and discomfort, chills, and fever for >1 month. She had a history of surgery for biliary stones but no history of hepatitis B, hepatitis C, or cancer. One month prior, the patient complained of recurrent pain and discomfort in the right upper abdomen without an obvious cause, accompanied by radiating pain in the right shoulder and back, chills, and fever without nausea, vomiting, or diarrhea. The patient’s routine blood and biochemical examination showed a white blood cell count of 9.54 × 10^9^/L, neutrophil percentage (%NETU) of 83.3%, C-reactive protein of 106.93 mg/L, alanine aminotransferase of 87U/L, aspartate aminotransferase of 102 U/L, alkaline phosphatase of 723 U/L, and glutamyl transpeptidase (γ-GT) of 708 U/L. Serum total bilirubin (TBil) 25.5 mmol/L, direct bilirubin (DBil) 14.9 mmol/L. Tumor markers showed CA-125 94.5 U/mL, CA19-9 485.44 U/mL, and CA15-3 22.2 U/mL. The AFP and CEA levels were within the normal ranges. Levels of glycated hemoglobin, urinalysis, and microscopy were normal. Tests for HBsAg, HBeAg e-antigen, and HCV virus were negative.

**Figure 1.**
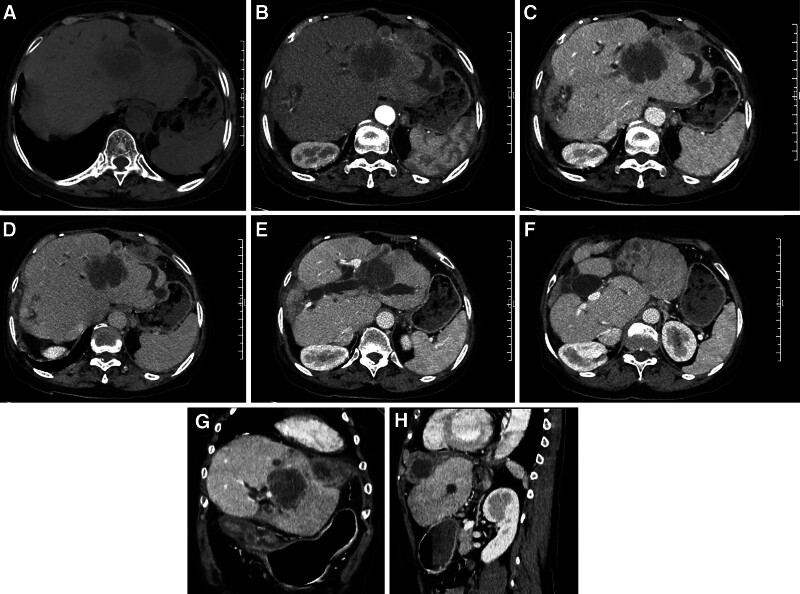
The patient’s CT scans. (A–D) Two space-occupying lesions are identified in the left lobe of the liver and subdiaphragmatic region, demonstrating low density on non-contrast CT. On contrast-enhanced imaging, the hepatic lesion displays ring-like enhancement with a central non-enhancing area and peripheral patchy mild enhancement. The subdiaphragmatic lesion exhibits thick-walled ring-like enhancement. (E) The lesion in the left lobe of the liver has encroached upon the bile duct and the left branch of the portal vein. (F) In the left lobe of the liver, there are also several small nodular ring-enhancing lesions. (G and H) The coronal and sagittal views reveal lesions in the left lobe of the liver and subdiaphragmatic region. CT = computed tomography.

**Figure 2.**
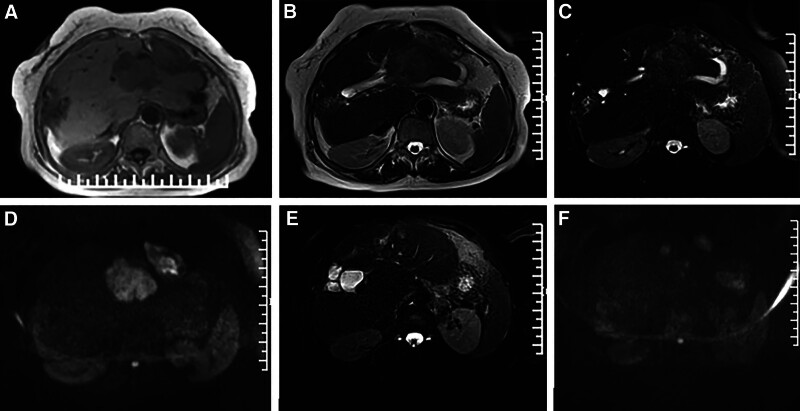
The patient’s MRI scans. (A–D) The MRI reveals a low signal on T1WI and a slightly higher signal on T2WI for the lesion in the left lobe of the liver, while it shows a slightly higher signal on DWI. As for the subdiaphragmatic lesion, it exhibits a low signal on T1WI, slightly higher to high signals on T2WI, and slightly higher to high signals on DWI. (E and F) On the T2-weighted fat-suppressed image, there are scattered nodular lesions observed in the left hepatic lobe, and the DWI sequence demonstrates mild diffusion restriction. DWI = diffusion-weighted imaging, MRI = magnetic resonance imaging, T1WI = T1-weighted imaging, T2WI = T2-weighted imaging.

Three abdominal computed tomography-enhanced scans revealed liver cirrhosis with multiple nodules and masses in the left lateral lobe. The largest lesion measured 5.2 cm × 4.7 cm × 8.0 cm, exhibiting lobulated margins. On computed tomography-enhanced scans, the edge of the maximum lesion showed persistent ring-like obvious enhancement, with a few patchy mild enhancement shadows within, demonstrating a “slow-in and slow-out" change (Fig. 1). Magnetic resonance imaging indicated slight hyperintensity on fat-suppressed T2-weighted imaging (T2WI) with patchy hyperintensity within the lesion, and diffusion-weighted imaging showed limited diffusion of water molecules (Fig. 2). Dilated bile ducts were observed around the largest lesion, with an unclear boundary between the dilated bile ducts and lesion. Invasion of the left branch of the intrahepatic portal vein was observed as a hypodense filling defect in some slices. Other small lesions in the left lateral lobe were partially adjacent to the largest lesion mentioned above, some scattered, and showed continuous mild ring enhancement on an enhanced scan. Additionally, a thick-walled irregular cystic solid mass with dimensions of 6.4 cm × 3.1 cm × 4.3 cm was found subdiaphragmatic in the prehepatic space. The sequence signal of the adipose-suppressed T2WI was similar to that of the lesions in the left lateral lobe. Computed tomography-enhanced scans showed obvious enhancement in the periphery of the lesion, but no enhancement in the center. In addition, closely related dilated bile duct shadows were observed around the lesion.

The patient underwent a left external liver lobectomy. During surgery, the diaphragmatic surface of the liver was attached to the diaphragmatic muscle. Multiple masses are observed in the left lobe of the liver. The section of the largest mass was pale yellow, and the adjacent intrahepatic bile duct and portal vein were invaded. Small lesions in the liver were partially dissected to reveal pus and partially solid nodules. A large amount of pus drained from the mass in the prehepatic space below the diaphragm, and solid lesions were observed in the remaining tissues.

**Figure 3.**
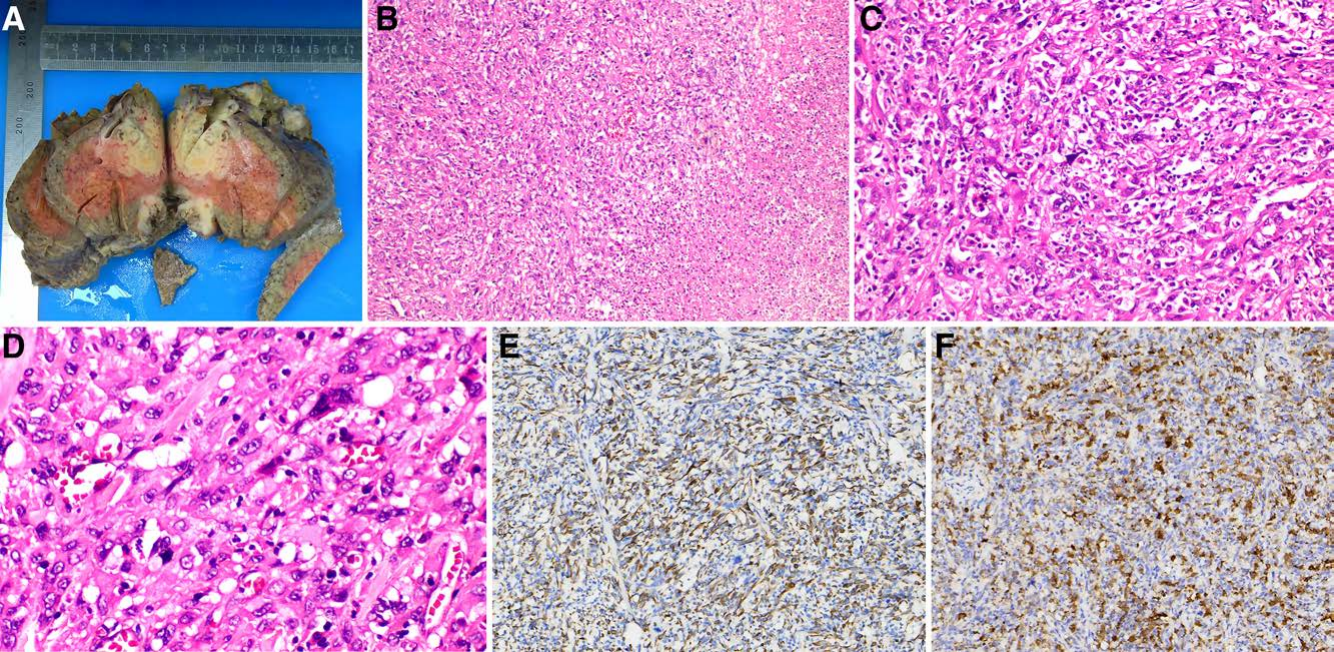
Tumor specimens and the results of pathological examinations. (A) Tumor specimens; (B–D) histological HE staining of the tumors at magnifications of 100×, 200×, and 400× respectively reveals extensive necrosis and a significant presence of atypical cells; (E and F) immunohistochemical staining at a magnification of 200× reveals positive expression of CK8/18 and GS.

Postoperative pathology revealed tumor cells in the lesions of the left outer lobe of the liver and in the prehepatic space below the diaphragm. These cells were arranged in sheets and exhibited obvious cell atypia, abundant cytoplasm, and a fusiform shape with widespread necrosis. Immunohistochemistry demonstrated that the tumor cells were positive for p-CK, GS, CK7, and CK8/18, partially positive for CK19 and CD10, and vascular-positive for CD34. However, they tested negative for Hepa, GPC-3, AFP, and HBsAg (Fig. 3). Based on these histological and immunehistochemical findings, we concluded that the pathological features were consistent with those of SHC.

The patient’s condition deteriorated after surgery, and hepatic encephalopathy developed 1.5 months post-operation, ultimately leading to death.

## 3. Discussion

The liver is susceptible to not only tumors, but also infections. Liver abscesses often present with fever and abdominal pain, with atypical symptoms and signs. In particular, when combined with liver tumors, symptoms and signs can be easily masked, leading to misdiagnosis.^[6]^ Bacteria can infect the central necrotic area of a primary hepatic tumor, leading to abscess formation.^[7]^

SHC is a relatively rare malignant tumor that grows rapidly, making it difficult for the blood vessels to meet the demands of the tumor, resulting in tissue liquefaction and necrosis.^[8]^ This growth pattern makes SHC more likely to cause nonspecific symptoms, such as fever and abdominal pain. Laboratory tests may show an increased white blood cell count, elevated C-reactive protein levels, and other inflammatory responses. Diagnosis can become even more challenging when SHC is complicated by abscess formation.^[9]^

In radiological diagnosis, rapid cell proliferation of the tumor leads to ischemia and necrosis, resulting in a central necrotic organization composed of inflammatory cells that increases arterial perfusion in the region. Therefore, SHC usually shows reinforcement around the large mass with central necrosis, which may be strengthened with or without a tumor capsule or intrahepatic/extrahepatic spread.^[5]^ These similarities to liver abscesses complicate differentiation.^[10]^

In clinical practice, both clinical and imaging departments often diagnose pyogenic liver abscesses based on nonspecific results that delay the diagnosis of malignant tumors. This delay results in patients not receiving timely treatment.^[11]^

In our case, the patient’s symptoms were nonspecific. Apart from a significant increase in Ca19-9, other laboratory tests did not contribute to the clinical suspicion of a tumor. Furthermore, a patient’s history of biliary calculi surgery may lead to misinterpretation in patients with biliary calculi and cholangitis. In the 3-phase enhanced scan, we observed thorough tumor necrosis, along with clear ring enhancement at the edge of the tumor and some mild patchy enhancements. There were no visible areas of improvement within the extensive tumor necrosis, consistent with the pathological tissue sections indicating extensive tumor necrosis. It has been reported that one direct difference between SHC and liver abscess is the ring-enhanced part of the lesion. Compared with the SHC, the abscess in the annular enhancement part was thicker.^[11]^ However, in our case, the lesion in the prehepatic space below the diaphragm also exhibited thick-walled enhancement similar to that of a liver abscess. On magnetic resonance imaging, both lesions showed slightly higher signal intensity on T2WI without clearly constrained dispersion sequences. This differs from liver abscess, which is characterized by a high T2WI signal accompanied by a clear water molecule diffusion limitation, which can aid identification. Additionally, patients with nodules in the left hepatic lobe and a few small nodules were pathologically reviewed as tumorous nodules, with some showing liquefaction necrosis. In conclusion, in this case report, we emphasize the importance of distinguishing SHC from liver abscesses when making a diagnosis based on imaging. When a liver abscess presents with low signal intensity on T1-weighted imaging and high signal intensity on T2WI on magnetic resonance imaging, along with ring-like enhancement at the edge of the lesion, and the presence of small bubbles and fluid within the lesion, these characteristics may aid in differentiation. Ultrasound-guided needle biopsy may be an effective method in challenging cases.^[11]^

Furthermore, this case shares numerous clinical and radiographic features with intrahepatic mass-forming cholangiocarcinoma. The patient presented with significantly elevated Ca19-9 levels, mass growth in the left lobe of the liver, and bile duct dilation around the mass. However, intrahepatic mass-forming cholangiocarcinoma typically present with painless jaundice at onset, and mass necrosis is uncommon. Radiographic findings revealed delayed centripetal enhancement, which differed from that in our case.^[12]^

We conducted a retrospective analysis of patients’ clinical data and imaging findings to elucidate their pathophysiological manifestations. The elevated inflammatory markers and clinical presentation of cold and fever in this patient may be attributed to intratumoral necrosis with an abscess, which is consistent with the findings of previous studies.^[7,13]^ Furthermore, elevated Ca19-9 levels in patients may be associated with intrahepatic bile duct invasion. We hypothesized that the transfer of tumor cells below the diaphragm is due to the obstruction of the left hepatic lobe lesions and ducts, leading to retrograde bile secretion and subsequent migration of tumor cells.

## 4. Limitations

Owing to the low incidence of SHC, our case reports were based on a single case. Therefore, our conclusions regarding radiological diagnosis may be limited. Nevertheless, in this case, we aimed to offer diagnostic insights for clinicians and radiologists.

## 5. Conclusion

We present a relatively rare case of SHC with subdiaphragmatic metastasis. The patient had not received antitumor therapy prior to diagnosis. Before surgery, the radiologist misdiagnosed the lesion as liver abscess. When imaging studies reveal intrahepatic space-occupying lesions with extensive necrosis and annular enhancement, consideration should be given to the possibility of SHC. Preoperative diagnosis of SHC with hepatic abscess is challenging. Further case-control studies on SHC and liver abscesses are necessary to enhance the accuracy of clinical and imaging diagnoses.

## Author contributions

**Conceptualization:** Chun-Yuan Yang, Xue-Ping Liu.

**Formal analysis:** Nan Lei, Xue-Wu Liu.

**Investigation:** Nan Lei, Xue-Wu Liu.

**Resources:** Xue-Wu Liu, Chun-Yuan Yang, Xue-Ping Liu.

**Supervision:** Nan Lei.

**Writing – original draft:** Xue-Wu Liu.

**Writing – review & editing:** Nan Lei, Chun-Yuan Yang, Xue-Ping Liu.
